# The influence of socio-demographic, psychological and knowledge-related variables alongside perceived cooking and food skills abilities in the prediction of diet quality in adults: a nationally representative cross-sectional study

**DOI:** 10.1186/s12966-016-0440-4

**Published:** 2016-10-26

**Authors:** Laura McGowan, Gerda K. Pot, Alison M. Stephen, Fiona Lavelle, Michelle Spence, Monique Raats, Lynsey Hollywood, Dawn McDowell, Amanda McCloat, Elaine Mooney, Martin Caraher, Moira Dean

**Affiliations:** 1 Centre for Public Health, School of Medicine, Dentistry and Biomedical Sciences, Queen’s University Belfast, Belfast, UK; 2 Diabetes and Nutritional Sciences Division, King’s College London, London, UK; 3 Department of Health and Life, Vrije Universiteit Amsterdam, Faculty of Earth and Life Sciences, Amsterdam, The Netherlands; 4 Department of Nutritional Sciences, School of Biosciences and Medicine, Faculty of Health and Medical Sciences, University of Surrey, Guildford, UK; 5 Institute for Global Food Security, School of Biological Sciences, Queen’s University Belfast, University Road, Belfast, BT7 1NN UK; 6 Food, Consumer Behaviour and Health Research Centre, School of Psychology, University of Surrey, Guildford, UK; 7 Department of Hospitality and Tourism Management, Ulster Business School, Ulster University, Coleraine, UK; 8 Department of Home Economics, St Angela’s College, Sligo, Ireland; 9 Department of Sociology, School of Arts and Social Sciences, City University of London, London, UK

**Keywords:** Cooking skills, Food skills, Diet, Healthy eating, Nutrition knowledge, Measurement, Eating Choices Index (ECI)

## Abstract

**Background:**

Interventions to increase cooking skills (CS) and food skills (FS) as a route to improving overall diet are popular within public health. This study tested a comprehensive model of diet quality by assessing the influence of socio-demographic, knowledge- and psychological-related variables alongside perceived CS and FS abilities. The correspondence of two measures of diet quality further validated the Eating Choices Index (ECI) for use in quantitative research.

**Methods:**

A cross-sectional survey was conducted in a quota-controlled nationally representative sample of 1049 adults aged 20–60 years drawn from the Island of Ireland. Surveys were administered in participants’ homes via computer-assisted personal interviewing (CAPI) assessing a range of socio-demographic, knowledge- and psychological-related variables alongside perceived CS and FS abilities. Regression models were used to model factors influencing diet quality. Correspondence between 2 measures of diet quality was assessed using chi-square and Pearson correlations.

**Results:**

ECI score was significantly negatively correlated with DINE Fat intake (*r* = -0.24, *p* < 0.001), and ECI score was significantly positively correlated with DINE Fibre intake (*r* = 0.38, *p* < 0.001), demonstrating a high agreement. Findings indicated that males, younger respondents and those with no/few educational qualifications scored significantly lower on both CS and FS abilities. The relative influence of socio-demographic, knowledge, psychological variables and CS and FS abilities on dietary outcomes varied, with regression models explaining 10–20 % of diet quality variance. CS ability exerted the strongest relationship with saturated fat intake (β = -0.296, *p* < 0.001) and was a significant predictor of fibre intake (β = -0.113, *p* < 0.05), although not for healthy food choices (ECI) (β = 0.04, *p* > 0.05).

**Conclusion:**

Greater CS and FS abilities may not lead directly to healthier dietary choices given the myriad of other factors implicated; however, CS appear to have differential influences on aspects of the diet, most notably in relation to lowering saturated fat intake. Findings suggest that CS and FS should not be singular targets of interventions designed to improve diet; but targeting specific sub-groups of the population e.g. males, younger adults, those with limited education might be more fruitful. A greater understanding of the interaction of factors influencing cooking and food practices within the home is needed.

## Background

The quality of the usual diet is not optimal in many parts of the world. In western countries, this can contribute to numerous chronic illnesses such as cardiovascular disease (CVD), cancers, and diabetes [1–5]. Multiple factors influence food choice and thus diet quality on a number of levels; for example, budget, resources, household structure and food availability (at a socio-economic level); taste preferences, food attitudes and identity, health motivations, nutritional knowledge and habitual behaviour (at an individual level) [6–9]. A series of reviews have suggested a relationship between individually-modifiable factors such as cooking skills (CS) and food skills (FS) and food choice and hence diet quality [10–12], indicating that greater cooking and food abilities are typically associated with better diet quality, such as increased consumption of fruit and vegetables (FV). Short [13] defined CS as a ‘set of mechanical or physical skills used in meal preparation’ including chopping, mixing, heating etc., but may also encompass conceptual and perceptual skills regarding the changes in food when cooked [13]. The term FS has grown in popularity as a means to recognise and measure the wider components of home meal production such as: meal planning, ingredient shopping, food budgeting, food safety and eating healthily. FS have been defined as the ability to ‘purchase, prepare and cook food materials using available resources, to produce well-balanced and tasty meals, appropriate to the age and needs of the individuals consuming them’ [14]. However, continued methodological difficulties in the definition and measurement of CS and FS are common, with few robust evaluations of interventions to improve these skills and their impact upon dietary behaviour, resulting in inconclusive findings on their effectiveness for improving diet [11, 12].

CS and FS interventions have grown in popularity across the UK, Europe and United States intended as a means to engage the public and to act as a conduit for improved dietary intakes [11, 15]. Public fascination with cooking and food in the media remains unwavering as the number of cooking and food-related entertainment programmes on television continues to grow [16, 17]; yet conversely, the use and consumption of convenience foods and pre-prepared products requiring fewer practical cooking skills to produce a meal has risen dramatically [18].

Existing data from the island of Ireland (IOI) is limited and focuses primarily on cooking skills, such as use of certain cooking techniques and confidence in cooking. Findings indicate that home food preparation is still commonplace, despite ambiguity over the number and types of cooking skills involved in meal production [19], that women retain greater responsibility for meal planning and food provision [19], that older persons are typically more confident in their cooking abilities than younger people [20], and, that socio-economic status may have a negative relationship with cooking ability [19, 21]. In addition, an increased nutrition knowledge has been shown to be associated with an improved diet quality [22, 23]. The most recently published national UK data came from the 2008–2009 wave of the National Diet and Nutrition Survey (NDNS) rolling programme which illustrated that respondents were reporting high confidence in cooking from basic ingredients and that associations with socio-demographic characteristics were scattered and inconsistent [19]. To our knowledge no other large-scale, nationally representative surveys have reported the relationship between perceived cooking and food skills abilities, food choice and diet quality alongside established factors such as socio-demographic factors, nutrition knowledge and psychological factors, such as health motivation and cooking identity, i.e., the degree to which someone identifies him or herself as a good cook. Therefore, we hypothesize that socio-demographic factors such as gender, age and socio-economic status will impact on diet quality in adults (H1). Further we expect those with higher nutrition knowledge will have a better diet quality. In addition we hypothesize that the possession of higher scores in psychological measures (being more health conscious, identifying as a cook and being more open to trying new foods) will influence better diet quality (H3) and finally those with a higher number of cooking and food skills would have a better diet quality (H4).

Using a quantitative approach, this research investigated the multiple and complex factors which influence diet quality in adults, including socio-demographic factors, nutrition knowledge and psychological factors alongside an assessment of perceived cooking and food skills ability (CS and FS ability respectively) in a nationally-representative survey conducted in Northern Ireland (NI) and the Republic of Ireland (ROI).

Two validated measures of diet quality suitable for use in large-scale quantitative surveys were employed: the DINE (Dietary Instrument for Nutrition Education), a food frequency questionnaire (FFQ) focusing on saturated fat and fibre intake [24]; and the ECI (Eating Choices Index), a brief four-item measure which aims to discriminate healthy and unhealthy eating behaviours [25]. Accurate dietary assessment is notoriously difficult in population-based quantitative research and FFQs are commonly employed to provide insight into dietary patterns however, these measures are often labour intensive with multiple self-report items. By contrast, the ECI is a brief four-item measure which has been validated against 5-d food diaries [25]. Therefore a secondary aim of this research was to investigate the extent of correspondence between DINE and ECI, providing further validation of the ECI as a brief indicator for dietary healthiness.

## Methods

### Design and sampling

Quota sampling stratified by Local Government District (LGD) on a proportionate basis using Probability Proportionate to Size (PPS) in NI, and stratified by Local Authority Area in ROI was used to obtain a nationally representative sample of 1049 adults aged 20–60 years from the Island of Ireland (NI and ROI), with tightly controlled quotas applied for: age; gender; socio-economic grouping; and, area of residence. In addition, respondents were only eligible if they cooked or prepared a main meal at least one or two times per week. The characteristics of these respondents can be seen in Table 1. Socio-economic groupings were created based upon the occupation of the highest earner in the household (developed by the ONS, UK Office for National Statistics) [26], and were classified as ABC1 (representing higher, intermediate, supervisory, clerical & junior managerial, administrative, professional occupations – higher socio-economic) versus C2DE (representing skilled, semi-skilled and unskilled manual occupations, unemployed and lowest grade occupations – lower socio-economic). Ethical approval for this study was obtained from Queen’s University Belfast Research Ethics Committee and research was conducted in accordance to the guidelines given in the Declaration of Helsinki. All participants were informed that by taking part in the survey they were giving consent for their data to be used. No personal details (name, address, date of birth) were recorded, only the sampling point. Participants were also made aware that that they could withdraw at any time.

**Table 1:** Sample Descriptives based upon ECI tertiles and DINE scores (low, medium and high)

	Overall Sample Mean (SD)/n (%)	ECI^a^				DINE Fat (saturated)				DINE Fibre			
		T1	T2	T3	*P* trend	Low	Medium	High	*P* trend	Low	Medium	High	*P* trend
		Mean (SD)/n (%)	Mean (SD)/n (%)	Mean (SD)/n (%)		Mean (SD)/n (%)	Mean (SD)/n (%)	Mean (SD)/n (%)		Mean (SD)/n (%)	Mean (SD)/n (%)	Mean (SD)/n (%)	
Age (*N* = 1049)	39.7 (11.8)	36.7 (11.7)	40.3 (11.8)	41.7 (11.6)	***	39.1 (12.0)	40.5 (11.8)	39.7 (11.7)	*	37.0 (11.7)	41.4 (11.8)	40.7 (11.5)	***
20–39 years (*n* = 545)	29.9 (5.7)	184 (33.8 %)	210 (38.5 %)	151 (27.7 %)	***	221 (40.6 %)	144 (26.4 %)	180 (33.0 %)	*	222 (40.7 %)	179 (32.8 %)	144 (26.4 %)	***
40–60 years (*n* = 504)	50.3 (6.4)	110 (21.8 %)	209 (41.5 %)	185 (36.7 %)	***	165 (32.7 %)	170 (33.7 %)	169 (33.5 %)	*	132 (26.2 %)	214 (42.5 %)	158 (31.3 %)	***
Gender													
Males	459 (43.8 %)	167 (36.4 %)	175 (38.1 %)	117 (25.5 %)	***	125 (27.2 %)	137 (29.8 %)	197 (42.9 %)	***	161 (35.1 %)	168 (36.6 %)	130 (28.3 %)	NS
Females	590 (56.2 %)	127 (21.5 %)	244 (41.4 %)	219 (37.1 %)	***	261 (44.2 %)	177 (30.0 %)	152 (25.8 %)	***	193 (32.7 %)	225 (38.1 %)	172 (29.2 %)	NS
Education													
No qualifications or compulsory level (*n* = 135)	135 (12.9 %)	51 (37.8 %)	52 (38.5 %)	32 (23.7 %)	***	51 (37.8 %)	33 (24.4 %)	51 (37.8 %)	**	57 (42.2 %)	48 (35.6 %)	30 (22.2 %)	NS
Secondary/further education (e.g., NVQ) (*n* = 656)	656 (62.5 %)	184 (28.0 %)	276 (42.1 %)	196 (29.9 %)	***	214 (32.6 %)	211 (32.2 %)	231 (35.2 %)	**	223 (34.0 %)	238 (36.3 %)	195 (29.7 %)	NS
University or higher (UG or PG degree) (*n* = 258)	258 (24.6 %)	59 (22.9 %)	91 (35.3 %)	108 (41.9 %)	***	121 (46.9 %)	70 (27.1 %)	67 (26.0 %)	**	74 (28.7 %)	107 (41.5 %)	77 (29.8 %)	NS
Socio-economic grouping													
ABC1 (*n* = 511)	511 (48.7 %)	125 (24.5 %)	188 (36.8 %)	198 (38.7 %)	***	199 (38.9 %)	168 (32.9 %)	144 (28.2 %)	**	161 (31.5 %)	188 (36.8 %)	162 (31.7 %)	NS
C2DE (*n* = 538)	538 (51.3 %)	169 (31.4 %)	231 (42.9 %)	138 (25.7 %)	***	187 (34.8 %)	146 (27.1 %)	205 (38.1 %)	**	193 (35.9 %)	205 (38.1 %)	140 (26.0 %)	NS
BMI (*n* = 760)	24.4 (4.2)	24.5 (4.2)	24.5 (4.3)	24.3 (4.1)	NS	24.2 (3.9)	24.8 (4.4)	24.4 (4.3)	NS	24.5 (4.0)	24.4 (4.3)	24.4 (4.3)	NS
Nutrition knowledge (*n* = 1049)	7.2 (2.2)	6.5 (2.4)	7.5 (2.0)	7.5 (2.2)	***	7.7 (2.0)	7.6 (2.1)	6.4 (2.3)	***	7.0 (2.4)	7.2 (2.0)	7.4 (2.2)	NS
Food and health consciousness (*n* = 1049)	3.8 (0.9)	3.5 (1.0)	3.8 (0.8)	4.1 (0.7)	***	3.9 (0.8)	3.9 (0.8)	3.6 (0.9)	***	3.6 (0.9)	3.8 (0.8)	4.0 (0.8)	***
Cooking identity (*n* = 1049)	24.5 (5.4)	21.7 (6.0)	24.9 (4.9)	26.3 (4.4)	***	24.7 (5.3)	24.8 (5.4)	23.9 (5.5)	*	23.3 (6.1)	24.7 (5.3)	25.5 (4.3)	***
Food neophilia (*n* = 1048)	10.5 (2.7)	9.5 (2.7)	10.6 (2.5)	11.2 (2.5)	***	10.7 (2.6)	10.8 (2.7)	9.9 (2.6)	***	10.3 (2.9)	10.4 (2.6)	10.7 (2.3)	NS
Meal prep. frequency (*n* = 1049)	1.7 (1.1)	1.3 (1.0)	1.7 (1.1)	1.9 (1.2)	***	1.6 (1.2)	1.7 (1.1)	1.7 (1.2)	NS	1.5 (1.2)	1.7 (1.1)	1.8 (1.1)	**
Cooking skills ability (*n* = 1049)	47.8 (29.3)	37.1 (29.0)	50.0 (28.9)	54.5 (27.7)	***	53.0 (27.9)	52.7 (31.4)	37.6 (26.1)	***	46.6 (33.6)	48.4 (27.0)	48.4 (26.8)	NS
Food skills ability (*n* = 1049)	45.8 (38.6)	34.4 (36.1)	47.7 (39.4)	53.5 (37.7)	***	47.0 (36.1)	55.3 (44.3)	36.0 (33.3)	***	46.5 (45.2)	44.5 (35.4)	46.7 (34.3)	NS
ECI score (*n* = 1049)	12.2 (2.9)	8.8 (1.4)	11.8 (0.8)	15.7 (1.5)	***	13.1 (3.0)	12.1 (2.7)	11.4 (2.8)	***	10.8 (2.8)	12.6 (2.7)	13/4 (2.7)	***
DINE Fat (*n* = 1049)	35.5 (13.0)	38.8 (13.3)	35.6 (12.0)	32.6 (13.4)	***	22.8 (5.0)	34.8 (3.0)	50.2 (9.0)	NS	34.1 (12.7)	35.2 (12.5)	37.6 (13.8)	NS
DINE Fibre (*n* = 1049)	34.6 (11.3)	29.3 (10.8)	34.4 (10.1)	39.5 (10.9)	***	33.1 (10.4)	35.2 (12.2)	35.8 (11.1)	NS	22.9 (5.1)	34.8 (3.1)	48.1 (7.2)	NS

*P* for trend: Chi square or ANOVA; NS: Non-significant; BMI; body mass index; *** Group difference is significant at the 0.001 level (2-tailed); ** Group difference is significant at the 0.01 level (2-tailed); * Group difference is significant at the 0.05 level (2-tailed)

^a^N.B. Higher ECI score reflects healthier choices; Higher DINE Fat score reflects higher fat intake; Higher DINE Fibre score reflects higher fibre intake

### Procedure

Sampling and all field data collection was conducted by the nationwide market research company SMR. Respondents were interviewed on a face-to-face basis in their own homes using Computer Assisted Personal Interviewing (CAPI); all interviewers were fully briefed before the commencement of fieldwork. Fieldwork on the survey was conducted between October and December 2014. Due to the nature of the sampling method, it was not possible to calculate specific response rates. However, where an interviewer was able to speak to a potentially eligible participant who declined to take part or who was not eligible, the reason was recorded. One hundred and 23 participants were unable to take part for the following reasons: dietary restrictions which interfered with food choices (*n* = 32); too busy (*n* = 26); not interested (*n* = 22); insufficient English language capability (*n* = 14); security concerns, such as preferring not to have an interviewer in the home (*n* = 5); never prepares or cooks a main meal (*n* = 6); outside age range of 20–60 years (*n* = 7); did not reside at address (*n* = 2); and, no reason given (*n* = 9). The survey was piloted via paper and pen with a range of individuals from varied backgrounds including students, employed, and unemployed adults (*n* = 40). Following the piloting phase only minor wording changes and amendments to the length of the overall survey were made by the research team, with an aim to reduce completion time and comprehension skills required. Following this, the survey was scripted and piloted in the field on two occasions prior to the main fieldwork, with the final version lasting an average of 38 min.

### Survey measures

The survey went through a number of stages of development. The first stage was informed by a review of the literature relating to influences upon food choice and diet quality, specifically examining the roles of cooking and food skills (see *McGowan et al. in press* [10]). A key component of the literature review was to critique the measurement of cooking and food skills within the existing literature. Interviews were also conducted with a number of experts who worked in the area of health promotion, including healthy eating and cooking and food skills education (*n* = 4). The interviews covered influences on diet quality, and specifically probed the role of cooking and food skills, and what these skills encompassed. These interviews highlighted the need for the use of clear terminology and for a relevant and accessible measure of cooking and food skills. Interviews were analysed using template analysis and important themes and content were used to guide the development of the survey items.

### Diet quality assessment

Two validated measures of diet quality were included in the survey. The ECI is a brief four-item measure to discriminate healthy and unhealthy eating choices [25] which covers frequency of consuming breakfast and two portions of fruit per day, and the type of bread and of milk typically consumed. The ECI has been shown to correlate with nutrient profiles consistent with a healthy diet in a five-day diet diary validation study [25]. ECI scores range from 4 to 20 with a higher score indicating healthier eating choices and for the purposes of analysis scores were classified into tertiles (T1, T2, T3) with T3 representing the highest or healthiest scores versus T1, the lowest scores. DINE is a brief dietary assessment questionnaire asking about the frequency of consumption of 19 different groups of foods which account for around 70 % of the fat and fibre in the typical UK diet [24], with emphasis on the main food sources of saturated fat. Foods which are similar in terms of nutrient content are combined and given a score proportional to the fat or fibre content of s standard portion size. DINE Fat and DINE Fibre scores are classified as follows: less than 30 low fat or fibre intake, 30 to 40 medium fat or fibre intake, and greater than 40 high fat or fibre intake. A high DINE Fat score therefore represents a high saturated fat intake, considered unhealthy in accordance with UK and ROI dietary guidelines; conversely a high DINE Fibre score represents a high fibre intake, considered healthy in accordance with UK and ROI dietary guidelines. DINE has been validated in a number of settings including primary care, and performed well when validated against 4-day diet diaries from a sample of factory workers [24].

### Predictor variables

Socio-demographic information included age, gender, education level and occupation of the highest household earner to enable socio-economic grouping [26]. Education level was categorised as follows: No qualifications or compulsory level only (i.e., schooling up to 15/16 years of age); Secondary/further education (e.g., National Vocational Qualification, NVQ); or, University level or higher (Undergraduate or Postgraduate degree). Participants were asked to report the number of other meals they typically prepared or cooked in the home each day aside from the main meal. This ‘meal preparation frequency’ variable was summed and treated as a continuous variable in analyses, ranging from 1 (preparing food or cooking typically only once per day) to 5 (preparing or cooking food on multiple occasions throughout the day). BMI (body mass index) was calculated (weight (kilograms)/height (m^2^)) from self-reported information from participants; height was reported in feet and inches or centimetres, and weight in kilograms or stones and pounds. All heights and weights were converted to kg and metres before conversion to BMI (Table 1).

Where possible the research team used existing reliable and valid instruments for all other components of the survey, including nutrition knowledge and health consciousness (Table 2). Cooking identity i.e., the degree to which someone sees his or herself as a good cook was assessed by 11 items based upon previous research with some minor adjustments [9, 27, 28]*.* This scale was also tested using factor analysis and subjected to reliability testing. Based on the results, the original 11 items were divided into cooking identity (seven items, one reverse coded) and food neophilia (three items); one item which did not load clearly onto either scale was deleted (Table 2).

**Table 2:** Description of predictor variables in the NI and ROI cohort (*n* = 1049)

Variable	No. of items	Range	Mean Score	SD	n	α
General nutrition knowledge^a^ * E.g. How many servings of FV a day do you think experts are advising people to eat as a minimum?*	12	0–12	7.20	2.20	1049	.65
Health Consciousness item^b^ * E.g. I am very particular about the healthiness of the food I eat* (R).	1	1–5	3.80	0.87	1049	-
Cooking identity^c^ * I am a good cook; Others view me as a good cook; I am a relatively better cook than my friends/family; I don’t consider myself to be a good cook (R); I get a sense of satisfaction from preparing and cooking meals; I am confident that whatever I cook will turn out well*; * I can time different elements of a dish to come together on time*.	7	7–35	24.45	5.39	1049	.88
Food neophilia^d^ * I like to try new foods; I like to try out new recipes; I would describe myself as a foodie (I’m interested in food and seek out food experiences)*.	3	3–15	10.47	2.67	1048	.74
Cooking skills ability^e^ * How good would you say you are at the following on a scale on 1–7 where 1 means very poor and 7 means very good…?* * 14 Items:* Chopping, mixing and stirring food; blending food; steaming food; boiling or simmering; stewing food; roasting food; frying/stir-frying food; microwaving food; baking cakes/bread/buns; peeling and chopping vegetables; preparing and cooking raw meat/poultry; preparing and cooking raw fish; making sauces and gravy from scratch; using herbs and spices to flavour food.	14	0–98	47.78	29.32	1049	.93
Food skills ability^f^ * How good would you say you are at planning meals ahead (e.g. for the day/week ahead) on a scale on 1–7 where 1 means very poor and 7 means very good?* * 19 Items:* planning meals ahead (e.g. for the day/week ahead); preparing meals in advance e.g. packed lunch, partly preparing a meal in advance; following recipes when cooking; shopping with a grocery list; shopping with specific meals in mind; planning how much food to buy; comparing prices before you buy food; knowing what budget you have to spend on food; buying food in season to save money; buying cheaper cuts of meat to save money; cooking more or double recipes which can be used for another meal; preparing or cooking a healthy meal with only few ingredients on hand; preparing or cooking a meal with limited time; using leftovers to create another meal; keeping basic items in your cupboard for putting meals together e.g. herbs/spices, dried/tinned goods; reading the best-before date on food; reading the storage and use-by information on food packets; reading the nutrition information on food labels; balancing meals based on nutrition advice of what is healthy.	19	0–133	45.82	38.64	1049	.94

^a^Parmenter & Wardle [41] (selected items taken from General Nutrition Knowledge (GNK) questionnaire with a range of response categories)

^b^ROIninen et al. [42] GHI single item used as indicator of General Health Interest (GHI) relating to food. Scores ranged from 1 = strongly agree, to 5 = strongly disagree [28]

^c^Devised and adapted from multiple sources including Keller et al. [27], Buckley et al. [28], Wansink [9] and by the research team. Scores ranged from 1 = strongly disagree, to 5 = strongly agree [9, 25, 26]

^d^Devised and adapted from Keller et al. [27], Buckley et al. [28] and by the research team. Scores ranged from 1 = strongly disagree, to 5 = strongly agree [25, 26]

^e^Devised and adapted from National Diet and Nutrition Survey (NDNS) Year 1, Barton et al. [43], Condrasky et al. [44], Wang & Worsley [50], Caraher, Dixon, Lang & Carr-Hill [21], Lyon, Syder, Flellstrom, et al. [20] and by the research team [21, 22, 41–43, 45]

^f^Devised and adapted from National Diet and Nutrition Survey (NDNS) Year 1 cooking items, Barton et al. [43], Brunner, van der Horst & Siegrist [46], Condrasky, [47], Morin et al. [48], Swindle, Baker, Auld [49] and by the research team [41, 42, 44, 46, 47, 50] (R) Reverse scored

Based upon the review of the literature, two new measures were produced for the present research for assessing cooking and food skills via self-report (see Table 2 for details). The cooking skills ability scale comprised 14 items and asked participants the following: ‘On a scale of 1 to 7 where 1 means very poor and 7 means very good, please say how good you are at…’ with options such as blending food, stewing food, roasting food, baking cakes/bread/buns, peeling and chopping vegetables and making sauces and gravy from scratch (Table 2). The food skills ability scale comprised the same as above but focused on wider food-related skills such as meal planning, preparing meals in advance, following recipes, shopping with a grocery list, comparing prices before buying, using leftovers to create another meal (Table 2). Participants were only asked to rate their CS or FS ability if they reported using each cooking or food skill after being shown a list. The cooking and food skills abilities assessment tool underwent rigorous development and psychometric testing (*unpublished data*). Reliability was high for both the CS and FS scales and principal component analysis illustrated that both components accounted for 73.5 % of the variance.

### Data analysis

All data were analysed using IBM SPSS Statistics Version 22 (IBM Corporation, 2013). Descriptive statistics were used to explore the data (means, standard deviations (SD), tertiles, etc.) in terms of socio-economic grouping and according to diet quality. T-tests, ANOVAs and crosstabs with Chi^2^ statistic were used to look for significant differences between the different levels of diet scores (ECI tertiles and DINE Fat and DINE Fibre high, medium, and low categories), and also the differences in cooking and food skills scores across demographic variables. Bivariate correlations using Pearson’s correlation coefficients were used to examine associations between socio-demographic, knowledge and psychological variables and diet measures. Pearson’s correlations were also used to examine the correspondence of the two diet quality measures (ECI and DINE Fat and DINE Fibre). Finally, hierarchical multiple regression analyses were conducted using the ECI score, DINE Fat and DINE Fibre as outcome (criterion) variables, predicted by socio-demographic variables, knowledge variables, psychological variables and cooking and food skills variables. A *p*-value of <0.05 was considered as statistically significant in all analyses and it was not deemed necessary to adjust for multiple testing within analyses reported here.

## Results

### Cooking Skills (CS) and Food Skills (FS) Ability

The CS and FS scales proved to have acceptable internal reliability (Cronbach’s alpha reported in Table 2, both > .90) and all items provided an acceptable two-factor structure as expected (cooking skills and food skills components), explaining 73.5 % of the variance.

Males scored significantly lower than females for CS (38.0 ± 27.6 versus 55.4 ± 28.4); older participants scored significantly higher than younger participants (43.5 ± 28.7 versus 52.4 ± 29.3); and those with no formal education or compulsory level education only, scored significantly lower than those with further or higher education (see Table 3).

**Table 3:** Differences in cooking skills (CS) ability and food skills (FS) ability on socio-demographic variables

	CS ability mean (SD)	*p* value	FS ability mean (SD)	*p* value
Age				
20–39 years (*n* = 545)	43.5 (28.7)	0.000	40.9 (36.5)	0.000
40–60 years (*n* = 504)	52.4 (29.3)		51.2 (40.2)	
Gender				
Males (*n* = 459)	38.0 (27.6)	0.000	35.5 (35.7)	0.000
Females (*n* = 590)	55.4 (28.4)		53.8 (39.0)	
Education				
No qualifications or compulsory level only (n = 135)	41.7 (29.0)	0.034	34.4 (31.7)	0.001
Secondary education/further education (e.g., NVQ) (*n* = 656)	48.7 (29.5)		47.7 (39.4)	
University or higher (UG or PG degree) (*n* = 258)	47.8 (29.3)		47.0 (39.1)	
Socio-economic grouping				
ABC1 (*n* = 511)	49.1 (29.4)	0.141	46.3 (38.7)	0.710
C2DE (*n* = 538)	46.5 (29.2)		45.4 (38.6)	

Overall, the sample mean was 45.8 (SD 38.6) for food skills; males scored significantly lower than females (35.5 ± 35.7 versus 53.8 ± 39.0); older participants scored significantly higher than younger participants (40.9 ± 36.5 versus 51.2 ± 40.2); and again, those with no formal education or compulsory level education only scored significantly lower on food skills ability (Table 3).

### Sample by ECI and DINE Classifications

Table 1 displays descriptive information for the sample broken down into low, medium and high scoring ECI tertiles, and DINE Fat and Fibre high, medium and low classifications. Older participants were significantly more likely to report healthier ECI scores (T2 and T3) and have a higher fibre intake (DINE), though the pattern was not clear for DINE Fat intake. The same pattern of results was noted for females. They also displayed significantly lower saturated fat scores compared to males. With regard to level of education and ECI scores, those with the lowest education scored poorly on the ECI (falling into T1 and T2 mainly) and were significantly more likely to report low levels of fibre intake. By contrast, those with a university education were significantly more likely to score in the highest ECI tertile (T3, i.e., making the healthiest choices) and have a significantly lower saturated fat intake. With regard to socio-economic grouping, those classified as ABC1 showed a graded pattern of response with few falling into the unhealthiest ECI range (T1) and most falling into T3. The greatest proportion of C2DE respondents (70 %) fell into unhealthier tertiles T1 and T2). The greatest proportion of ABC1 participants scored in the lowest range of saturated fat intake, whereas the greatest proportion of C2DE participants scored high on saturated fat intake. There was no significant difference between the socio-economic groupings in relation to fibre intake.

Those with the greater nutrition knowledge reported significantly healthier food choices as measured by the ECI (*p* < 0.0001), and those consuming the least amount of saturated fat (DINE) had significantly greater nutrition knowledge (*p* < 0.0001). Where participants indicated a greater concern for the healthiness of food this was reflected in their dietary intake, as the average score for food and health concern was greatest in the healthiest ECI tertile (T3); lowest in the high saturated fat intake group; and, highest in the high fibre intake group, in line with current dietary recommendations. There was an association between cooking identity and diet quality, with those who saw themselves as good cooks and those with greater food neophilia (openness and interest in food) displaying healthier ECI scores, lower saturated fat and higher fibre scores). Daily meal preparation frequency showed a significant trend towards a healthier dietary profile for ECI scores (*p* < 0.0001) and DINE-fibre score when cooking on more than just one occasion per day (*p* < 0.0001). Cooking skills and food skills both showed the same pattern of responses for ECI scores in that those classified into the healthiest ECI tertile reported the greatest cooking and food skills abilities. In addition, those classified as having a high saturated fat intake scored (DINE) significantly lower on both cooking skills and food skills ability.

BMI did not differ significantly across categories of diet quality.

### Correspondence between DINE and ECI

Descriptive statistics for the three dietary measures illustrate a clear pattern; DINE Fat intake scores were highest in the lowest (unhealthiest) ECI tertile and reduced significantly as the ECI scores increased (Table 1). The reverse pattern was observed for fibre intake, with the highest DINE Fibre scores noted in the healthiest ECI tertile. Correspondence was further illustrated by correlations between the diet measures; ECI score was significantly negatively correlated with DINE Fat intake (*r* = -0.24, *p* < 0.001), and ECI score was significantly positively correlated with DINE Fibre intake (*r* = 0.38, *p* < 0.001).

The association between ECI tertiles and DINE Fat and DINE Fibre respectively was examined using crosstabs with the chi^2^ statistic; this illustrated that the greatest proportion of participants classified in the lowest ECI tertile (unhealthiest) were also those consuming the greatest amount of saturated fat (*n* = 130); and those classified in the highest ECI range, T3 (healthiest) were also those consuming the least amount of saturated fat according to the DINE (*n* = 162), χ^2^ (4) = 41.3, *p* < 0.001. Similarly, the greatest proportion of participants scoring poorly in the ECI (i.e., lowest tertile) were most likely to have a low fibre intake as measured by the DINE Fibre score (*n* = 164); and those in the healthiest ECI tertile were most likely to be categorised as having a high fibre intake (*n* = 145), χ^2^ (4) = 123.8, *p* < 0.001 (data not shown here, see Supplementary Material for Tables 1 and 2).

### Associations between cooking skills and food skills ability and diet quality

A positive correlation was found between cooking skills ability (CS) and food skills ability (FS) (*r* = 0.76, *p* < 0.001) indicating the scales measure highly related components (though distinct as shown by principal components analysis, *data not presented here*). In relation to cooking and food skills abilities and diet outcomes, the same pattern of results was found for both cooking skills and for food skills and two of the diet quality indicators in that both showed a positive correlation with the ECI (cooking skills ability and ECI *r* = 0.26, *p* < 0.001; food skills ability and ECI *r* = 0.19, *p* < 0.001); both scales also showed a negative correlation with DINE Fat scores (cooking skills ability and DINE Fat *r* = -0.22, *p* < 0.001; food skills ability and DINE Fat *r* = -0.11, *p* < 0.001).

### Predictors of diet quality

In the regression analysis predicting ECI scores (Table 4), numerous socio-demographic, knowledge-related, and psychological variables remained significant in the final model including: age, gender, education level, socio-economic status, nutrition knowledge, food and health consciousness, cooking identity and meal preparation frequency, with food and health consciousness having the highest predictor value (β = 0.172, *p* < 0.001). Neither cooking skills ability nor food skills ability added any variance explained to the final model which overall accounted for 19.5 % of the variance (R^2^) in ECI dietary score (Model F [11,1047] = 22.782, *p* < 0.001).

**Table 4:** Hierarchical multiple regression analyses for ECI, DINE Fat and DINE Fibre outcomes predicted by socio-demographic, knowledge, psychological variables and cooking and food skills variables

	ECI		DINE Fat		DINE Fibre	
	B (SE)	β	B (SE)	β	B (SE)	β
Model 1: Socio-demographic	R2 change = 0.089***		R2 change = 0.072***		R2 change = 0.027***	
Age	0.660 (0.174)	0.112***	1.229 (0.773)	0.047	2.442 (0.703)	0.108**
Gender	0.523 (0.176)	0.088**	-5.560 (0.780)	-0.212***	-1.121 (0.709)	-0.049
Education level	0.219 (0.081)	0.088**	-0.682 (0.357)	-0.062	0.747 (0.325)	0.079*
SES	-0.420 (0.182)	-0.071*	0.324 (0.810)	0.012	-0.505 (0.736)	-0.022
Model 2: Knowledge	R2 change = 0.028***		R2 change = 0.050***		R2 change = 0.004*	
Nutrition knowledge	0.096 (0.042)	0.071*	-1.134 (0.187)	-0.191***	0.094 (0.170)	0.018
Model 3: Psychological	R2 change = 0.073***		R2 change = 0.022***		R2 change = 0.054***	
Food and health consciousness	0.583 (0.112)	0.172***	-1.699 (0.497)	-0.113**	2.205 (0.452)	0.170***
Cooking identity	0.084 (0.023)	0.154***	0.473 (0.100)	0.195***	0.505 (0.091)	0.242***
Food neophilia	0.036 (0.042)	0.033	-0.557 (0.189)	-0.114**	-0.417 (0.171)	-0.099*
Model 4: Cooking and food skills	R2 change = 0.006		R2 change = 0.046***		R2 change = 0.017***	
Meal prep frequency	0.184 (0.078)	0.072*	1.766 (0.348)	0.156***	0.884 (0.317)	0.090**
Cooking skills ability	0.004 (0.005)	0.040	-0.132 (0.021)	-0.296***	-0.043 (0.020)	-0.113*
Food skills ability	-0.005 (0.003)	-0.070	0.056 (0.015)	.166***	-0.015 (0.014)	-0.052
Final Model R^2^	0.195***		0.190***		0.101***	

*SES* socio-economic status*, ECI* Eating choices Index*, DINE* Dietary instrument for nutrition education. ***Group difference is significant at the 0.001 level (2-tailed); **Group difference is significant at the 0.01 level (2-tailed); *Group difference is significant at the 0.05 level (2-tailed)

In the regression analysis predicting DINE Fat intake all models were significant with gender, nutrition knowledge, food and health consciousness, cooking identity, food neophilia, meal preparation frequency, cooking skills ability and food skills ability all contributing significantly to the final model, with the strongest contribution coming from cooking skills ability (β = -0.296, *p* < 0.001) where greater cooking skills were associated with lower fat intake. The final model accounted for 19.0 % of the variance (R^2^) in DINE Fat intake score (Model F [11,1047] = 22.038, *p* < 0.001).

Finally, in the regression analysis predicting DINE Fibre intake all models were significant with age, education level, food and health consciousness, cooking identity, food neophilia, meal preparation frequency and cooking skills ability all significantly contributing to the final model, with the strongest contribution from cooking identity (β = 0.242, *p* < 0.001). Here, a greater cooking identity was as associated with increased fibre intake although greater perceived CS ability was associated with lower fibre intake. The final model accounted for 10.1 % of the variance (R^2^) in DINE Fibre intake score (Model F [11,1047] = 10.623, *p* < 0.001).

All three regressions were re-run substituting the variables CS and FS ability with CS competence and FS competence (i.e. the total number of CS or FS the participant reported using before rating their ability on each, respectively). The patterns of dietary results was unchanged for ECI and DINE Fat scores, although the regressions explained less variance. For DINE Fibre the results were minimally different in that CS competence (versus CS ability) did not contribute significantly to the model with less variance was explained overall.

## Discussion

This research investigated the multiple and complex factors which influence diet quality in adults in a nationally-representative survey conducted in Northern Ireland (NI) and the Republic of Ireland (ROI). This was measured by two validated dietary instruments (ECI and DINE), and influencing factors included socio-demographic factors, nutrition knowledge and psychological factors alongside an assessment of perceived cooking and food skills ability (CS and FS ability respectively).

### Interpretation and implication of results

This cross-sectional investigation into multiple and complex determinants of diet quality in a nationally representative sample of adults aged 20–60 years on the Island of Ireland (NI and ROI) revealed a number of interesting findings not previously reported.

Diet quality in this sample was comparable to that found in previous research which reported findings from a sample of over 2000 adults aged 43 years (mean BMI 24.8 ± 3.9, 49 % male) participating in the Medical Research Council National Survey of Health and Development (NSHD; 1946 British birth cohort) [25]. The mean values for the DINE also showed a similar pattern to previous research conducted 20 years earlier) [24]. The mean DINE Fibre score for the present sample was also comparable to previous research although with less highly educated participants [24]. It was of interest that BMI did not differ across the diet quality classifications for either the ECI or the DINE; this raises the issue that diet quality does not appear to affect weight status in this sample and perhaps other factors are of more importance for BMI, such as portion size, total energy intake, eating pattern, and physical activity levels. Indeed, recent research has highlighted the positive association between BMI and total energy intake in adolescents in the UK based on NDNS data [29]. The survey did not include a measurement of physical activity due to the length and time of the survey and should be investigated in future research to see whether physical activity impacts upon BMI and the differing dietary factors in this context. In addition there may have been measurement issues with BMI classifications or response bias due to self-reported BMI.

In relation to cooking and food skills ability, previous research has indicated an association between cooking and food skills (CS and FS respectively) and diet quality [10–12], with better skills corresponding to better quality. Only partial support for such a relationship was provided here. Descriptive statistics and bivariate correlations revealed interesting patterns across the diet quality indices; for example in correlation analyses, the psychological variables relating to cooking identity, food neophilia and health consciousness were most strongly associated with ECI scores (i.e. healthier choices), followed by perceived cooking and food skills ability. A similar pattern was found for fibre intake, where food and health motivations and cooking identity was significantly associated. However, a different pattern again was observed for saturated fat intake as measured by the DINE, as the strongest associations came from nutrition knowledge and cooking skills ability, highlighting a role for knowledge and self-efficacy i.e. perceived cooking ability, in determining fat intake. The link with nutrition knowledge supports previous research which has shown that greater nutrition knowledge is related to consumption of a diet more closely aligned to healthy eating guidelines in the UK [30], and intervention studies have shown that dietary fat intake can be reduced through participation in cooking classes [31].

The multivariate analyses revealed a more complex picture however; perceived CS ability independently explained variance in saturated fat intake as measured by the DINE over and above gender, nutrition knowledge and psychological variables such as cooking identity and openness to food (*R* ^2^ = 19 %). Here, CS ability provided the strongest contribution to the model in that those reporting greater perceived CS abilities consumed less saturated fat, supporting findings from previous intervention studies which targeted dietary improvements through Mediterranean diet cooking classes [31]. However, a positive association was noted between perceived FS ability and saturated fat intake which is more difficult to explain; perhaps those with greater food skills are those who are more elaborate or indulgent cooks, who value taste over nutritional qualities as shown in previous research [32]. The present model explains a much greater proportion of variance than a previous study which included only socio-demographic variables and nutritional knowledge to explain 11 % (R^2^ adj.) of the variance in DINE Fat scores [30].

The final regression model produced for fibre intake explained the least amount of variance of the three diet indices (*R* ^2^ = 10.1 %) with cooking identity making the strongest contribution, in that those identifying as better cooks were more likely to have a high fibre intake. Food and health consciousness was also a strong predictor for fibre intake although interestingly nutritional knowledge did not exert a strong influence in the multivariate regressions, despite its association with fruit and vegetable intake (high fibre) in previous research [30]. CS ability appeared to have a negative association with fibre intake in that greater perceived CS ability was associated with lower fibre intake which seems to oppose the findings for saturated fat, where greater CS led to a healthier dietary pattern. Again, the reasons for this are unclear, but levels of fibre intake were low for the sample overall and perhaps indicates that the nutritional messages around fibre have not permeated well in comparison to those about saturated fat intake, regardless of perceived CS or FS abilities. Fibre is often not a key element of front-of-pack labelling, meaning the general population may be less aware of the targets for a healthy diet. This is supported by findings from the National Adult Nutrition Survey in Ireland (2011) by IUNA (Irish Universities Nutrition Alliance) which also reported low fibre levels. In fact over 80 % of adults were reported as failing to meet the European Food Safety Authority recommendation of 25 g fibre per day. It could also indicate a role for seasonality of diet, as the winter period when this survey was conducted might inherently encourage greater saturated fat intake and lower fibre intake (e.g., fruit and vegetables).

CS and FS did not add independent variance explained to the ECI regression, where instead the strongest contribution to the model came from food and health consciousness, in that those who were more particular about choosing healthy foods were indeed more likely to make healthier choices which has been shown in previous research [6]. Based on these collective findings it would appear that perceived CS and FS abilities have differential effects on dietary patterns, with greater CS abilities most likely to influence saturated fat intake. Attempts to target CS and FS abilities via public health interventions may only exert a small influence upon dietary quality, although it may be the case that specific sub-groups of the population could benefit more from this approach where CS and FS abilities were significantly lower, i.e., younger age groups, males and those with little/no formal education. This echoes recent findings by Adams and colleagues [19] in their examination of CS in the UK via the National Diet and Nutrition Survey (NDNS) conducted in 2008. Despite the varied influence of CS and FS abilities on diet quality in this sample it is worth noting that recent research with young adults in America found that preparing meals at home was associated with better diet quality outcomes, such as greater fruit and vegetable intakes [33] and more importantly, meal preparation and home cooking behaviours showed evidence of tracking throughout young adult life [34]. Perhaps CS and FS abilities warrant greater attention in the early educational years to equip people with the skills necessary for future healthy cooking habits. Furthermore, given the important role cooking identity played in all dietary outcomes it might also be worthwhile targeting this within any future interventions and theoretical frameworks such as the theory of planned behaviour or social cognitive theory may provide suitable insertion points for attitudinal and identity change.

### ECI and DINE

There was good correspondence between the two previously validated measures of dietary assessment, with the majority of those scoring high on the ECI (healthier) also scoring low on saturated fat intake as measured by the DINE. Similarly, those scoring in the lowest category of the ECI (e.g., T1, unhealthier choices) scored high on saturated fat intake and low on fibre intake. Given that the ECI is a brief 4-item measure this provides further validation of its measurement abilities and support for its use in large-scale surveys. A limitation to consider however for the DINE relates to the lack of portion size information – although neither measure quantifies portion size, the DINE bases its weighting on portion size information from over 20 years ago [24] which may now be inaccurate based on current trends [35, 36]. It may be the case that future updates to the DINE could assess any population changes in portion sizes for the foods covered and reflect this in the scoring accordingly. The authors of the ECI suggest that not accounting for portion size is a strength of their tool given the difficulties with accurate quantification of portion size. In addition it needs to be noted that both measure used in this study were self-reported proxies for diet intake as many measures of dietary assessment are. It would be interesting to confirm these findings in other large observational and prospective studies as using direct dietary intake measures may not be feasible in large samples for numerous reasons including cost.

### Strengths, limitations and future research directions

Strengths of this research include diet quality and cooking and food skills data collection from quota-controlled nationally representative sample of adults living in Northern Ireland and the Republic of Ireland; despite screening for those who prepared or cooked a main meal at least once or twice per week the overall sample closely matched that of recent census estimations for both NI and ROI [37, 38]. Further, for the use on a large scale survey basis the CS and FS abilities scales were found to be reliable, valid and easy to use after extensive development and testing, which is beneficial for future research as this has been a problematic measure in previous research.

Previous studies have noted response bias and patterns of socially desirable responses with self-rated confidence items in that participants tend to rate themselves as highly confident across items, even when they may not have any practical experience with the skills in question. This was overcome in this survey by using a method of first asking the participant to look at the list of CS and FS and say which ones they used. For those skills for which participants had practical experience, they then rated their ability from very poor to very good. This meant that mean CS and FS values were not over-inflated. A practical measure of CS and FS instead of perceived measures would be interesting to use in future studies in comparison to a dietary intake measure, to confirm some of these findings, however, this may not be feasible on such a large scale. In addition longitudinal experimental studies could help to assess the impact of cooking skills on dietary intake, for example, a cooking and food skills intervention with the aim of improving diet quality and dietary intake with a baseline practical measure of cooking skills and dietary intake and also multiple follow-up points to assess the retention of cooking skills level and diet quality. Future research should aim to devise internationally recognised practical measures for cooking and food skills, which has been difficult due to cultural differences in cooking skills used. An agreed standard measure for both cooking and food skills would allow for greater comparisons between studies and improve this growing research area. Further, although some level of social desirability was addressed, this remains a problem in research of this nature. The positive correlation seen between health consciousness and diet quality could be noted as an example of social desirability. Future research may attempt to address this by targeting specific subpopulations with lower levels of health consciousness and a range of cooking abilities to assess their diet quality. A further limitation to study, as with most cross-sectional research is self-report which depends on respondent memory, impacting on the accuracy of responses.

A limitation of the findings was the relatively low amount of variance explained for fibre intake as measured by the DINE (10.1 %) compared to fat intake and ECI scores; however, fibre intake is below average in the UK [39] with fibre intake guidelines less well known in multiple countries including the US and Europe [40] so perhaps this limited the findings. However, for the ECI and fat intake, approximately 20 % variance being explained for both is higher than in previous similar studies [25, 30]. The present study was much more comprehensive in its assessment of the influence of demographic factors, knowledge-related factors, psychological factors as well as perceived cooking and food skills abilities, which may have influenced the results.

## Conclusion

Greater perceived CS and FS abilities are not conclusively associated with healthier dietary choices (as measured by the ECI) or dietary patterns (as measured by the DINE) given the myriad of other factors implicated such as socio-demographics (age, gender, education); nutritional knowledge; and psychological factors such as food and health motivations and cooking identity. However, CS and FS abilities do appear to have a differential impact upon aspects of the diet, most notably in relation to saturated fat, where greater perceived CS are associated with reported reduced saturated fat intake. Findings from this research suggest that CS and FS should not be singular targets of interventions designed to improve diet quality, and that such interventions should also focus on a wide range of knowledge- and psychological-related factors such as cooking identity and health motivations. Furthermore, targeting the CS and FS abilities of specific sub-groups of the population like males, younger adults and those with limited education, alongside other diet-related determinants reported here, might be more fruitful and cost-effective than a population- or community-wide approach. A greater understanding of the interaction of multiple factors influencing cooking and food practices within the home and their relationship with diet quality is needed. It is also worth noting that using a brief four-item measure such as the ECI to capture dietary choices may be sufficient in place of longer dietary assessment measures, given the correspondence with the DINE and the ECI’s ability to predict dietary outcomes.

## Abbreviations

CAPI: Computer Assisted Personal Interviewing
CS: Cooking skills
CVD: Cardiovascular disease
DINE: Dietary Instrument for Nutrition Education
ECI: Eating Choices Index
FS: Food skills
FV: Fruit and vegetables
IOI: Island of Ireland
NDNS: National Diet and Nutrition Survey
UK: United Kingdom
